# Suprapubic Catheter Migration: A Review of a Rare Complication

**DOI:** 10.1155/2021/8816213

**Published:** 2021-01-05

**Authors:** Amr Elmoheen, Mahmoud Saqr, Waleed Salem, Khalid Bashir, Ayman Hagras

**Affiliations:** ^1^Emergency Department, Hamad Medical Corporation, Qatar; ^2^QU Health, College of Medicine, Qatar University, Qatar; ^3^Urology Department, College of Medicine, Tanta University, Egypt

## Abstract

**Background:**

Suprapubic catheter migration to the vesicoureteral junction is an unusual complication, causing an obstruction that led to hydronephrosis and dilation of the pelvicalyceal system. *Case presentation.* A 30-year-old man with a suprapubic catheter (SPC) that was inserted one month before this current Emergency Department (ED) visit had severe left flank pain for 48 hours. The SPC was inserted in the context of urethral injury after falling astride. Point-of-Care Ultrasound (POCUS) showed a semifilled urinary bladder and moderate hydronephrosis on the left side. A computed tomography scan (CT scan) of the abdomen was performed and showed migration of the suprapubic catheter's tip into the left vesicoureteral junction, causing ureteral obstruction dilation of the ipsilateral pelvicalyceal system. The suprapubic catheter was changed in the ED, causing relief of symptoms, and the patient was referred to the urology department for follow-up. It was uneventful on the follow-up from the SPC clinic.

**Conclusions:**

This case report describes a rare complication of migration of the suprapubic catheter to the vesicoureteral junction causing acute ureteral obstruction and hydronephrosis.

## 1. Introduction

Suprapubic catheter drainage is a widely used urological procedure in patients with neurogenic bladder and acute retention of urine with failed urethral catheterization or in patients in whom the placement of a urethral catheter is contraindicated as urethral trauma [1]. The complication rate associated with cystotomy varies from 1.6 to 2.4% [2]. A poorly reported complication but with a significant impact on the quality of life of patients is catheter migration, which should be considered in patients with acute urine retention, obstructive urinary symptoms, low back pain, or impaired renal function [3]. This review describes an unusual complication of migration of the suprapubic catheter to the vesicoureteral junction, causing an obstruction that led to hydronephrosis and dilation of the left pelvicalyceal system.

## 2. Patient Information

A 30-year-old man with a suprapubic catheter (SPC) that was inserted one month before this current Emergency Department (ED) visit had severe left flank pain for 48 hours. The SPC was inserted in the context of urethral injury after falling astride. He did not report any fever, changes in urine coloration, nausea, vomiting, or diarrhea. The suprapubic catheter was changed five days before this current ED presentation, and the patient reported some resistance and difficulty during the exchange. Physical examination reveals only granulomatous tissue with pus in the suprapubic catheter port; however, there is no pus in the catheter track. The rest of the general and systemic examination was within normal parameters.

## 3. Investigations

In order to rule out the presence of a urinary tract infection, a urine test was performed that reveals a high white blood cell count (28 *μ*L). Laboratory tests reported leukocytosis (13900/*μ*L), elevated absolute neutrophil count (10300/*μ*L), hyponatremia (132 mmol/L), hypochloremia (93.0 mmol/L), and normal kidney function (creatinine, 77 *μ*mol/L).

Bedside Point-of-Care Ultrasound (POCUS) showed a collapsed urinary bladder and moderate to severe hydronephrosis on the left side, and the balloon of the catheter in the bladder cannot be observed.

**Figure 1:**
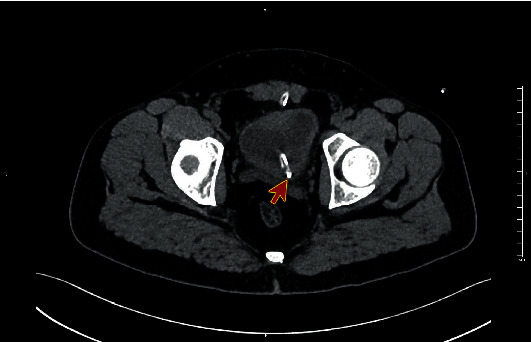
Computed tomography (CT) abdomen, axial view showing the tip of the suprapubic catheter inserted at the left ureterovesical junction (red arrow).

**Figure 2:**
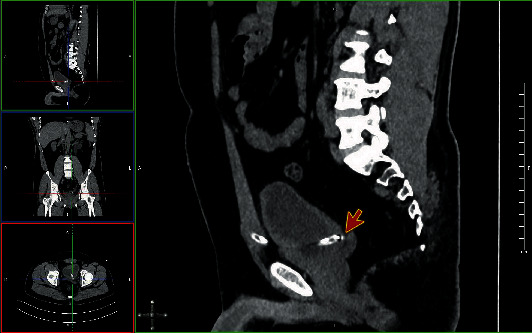
Computed tomography (CT) abdomen, sagittal view showing the tip of the suprapubic catheter inserted at the left ureterovesical junction (red arrow).

**Figure 3:**
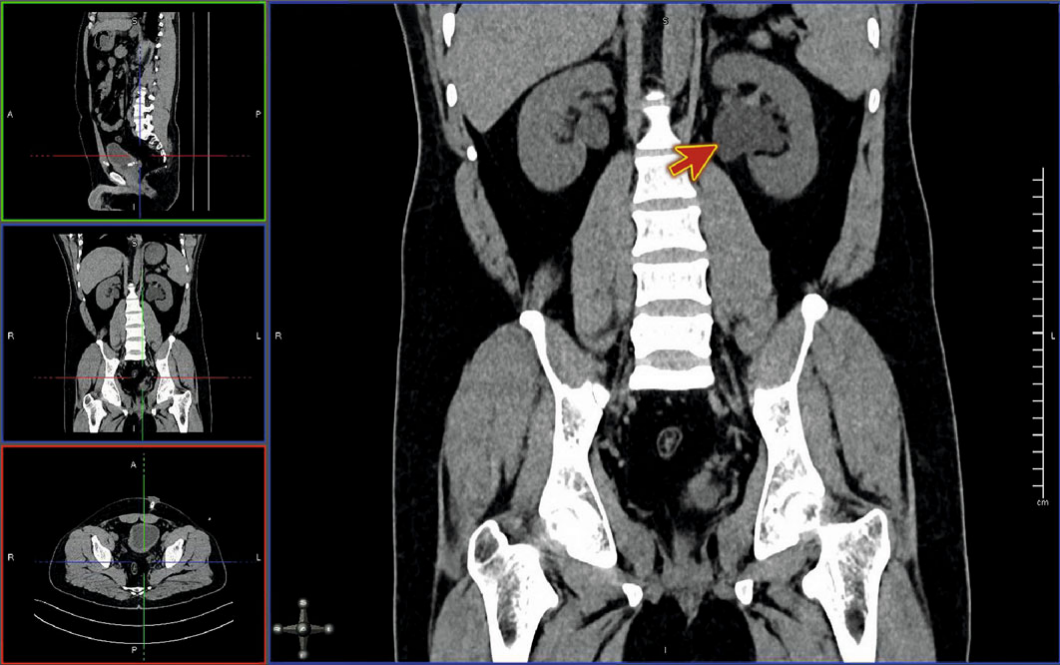
Computed tomography (CT) abdomen, coronal view showing moderate dilatation of the left pelvicalyceal system (red arrow).

Computed tomography (CT) abdomen was performed, showing the tip of the suprapubic catheter inserted at the left ureterovesical junction causing obstruction the ureter (Figures 1 and 2) and moderate dilatation of the left pelvicalyceal system (Figure 3). This finding confirms the diagnosis of catheter migration.

## 4. Treatment

The emergency physician replaced the old suprapubic catheter with a new 16 Fr catheter. Its proper placement was confirmed by ultrasound, and a sample was taken for urine culture. After replacing the catheter, the patient drained 1200 mL of clear urine over 3 hours. At the time of home discharge, the patient was in good general condition and pain-free, and the catheter was draining clear urine. Outpatient treatment with ciprofloxacin 500 mg orally every 12 hours for seven days, paracetamol 1 gram orally every 8 hours for seven days, and outpatient follow-up with urology service were arranged.

## 5. Outcome and Follow-Up

After performing a combined cystourethrogram, that revealed short urethral stricture (less than 5 mm), a visualized internal urethrotomy was done after two weeks. During the cystoscopy, the left ureteral orifice was slightly dilated than the right one. The suprapubic catheter was removed, and an 18 Fr silicone urethral catheter was fixed. The patient had a regular follow-up with the urology outpatient services for three months. He did not report any new complications later on.

## 6. Discussion

The placement of a suprapubic cystotomy catheter is a frequent procedure used in urology, especially in patients with neurogenic bladder who require long-term bladder drainage. Suprapubic cystotomy is also indicated in cases of trauma or urethral pathology that prevent the placement of a transurethral catheter. The most frequent complications associated with cystotomy are bacteriuria, bleeding, and bladder stones [4]. Other complications such as intestinal perforation, enterocutaneous fistulas (ECF), neoplastic changes in the urinary bladder, and migration of the catheter into the ureter have also been reported [5, 6]. However, multiple meta-analyses have shown that bladder drainage using a suprapubic catheter is associated with a lower rate of complications compared to transurethral catheter drainage.

The migration of the suprapubic catheter to the vesicoureteral junction is a rare complication. The patients at greatest risk are those who have a permanent catheter and/or require long-term bladder drainage. This is because the bladder of these patients tends to contract, modifying the anatomical relationship between the bladder neck and the ureterovesical junction, which makes it easier for the catheter to enter the ureter. Furthermore, these patients require a regular change of the catheter; therefore, there is a greater probability that the catheter will inadvertently be inserted into the ureter. Patients with neurogenic bladder usually present ureterovesical reflux, which also predisposes to this complication [7].

In 1987, Borrero et al. reported a 35-year-old paraplegic man with suprapubic catheter migration diagnosed by intravenous venogram [8]. In 2010, Dangle Pankaj et al. described another case of suprapubic catheter migration to the left ureter that led to obstructive uropathy and hydronephrosis in a patient with a neurogenic bladder [9]. In 2011, Singh et al. defined a case of suprapubic migration due to a gaping ureteric orifice in a 27-year-old man with traumatic bulbomembranous urethral stricture [10]. In 2016, Luo et al. reported three cases of accidental suprapubic catheter placement at the vesicoureteral junction in patients with neurogenic bladder and/or voiding dysfunction [7]. In the same year, Shuaibin et al. reported on the case of an elderly male patient with senile dementia whose suprapubic catheter had migrated into a previously normal nongaping ureter [11].

This paper presents the case of a male patient, 30-year-old, with a history of urethral trauma (falling astride) that is managed by suprapubic catheter insertion one month before his current ED presentation to be prepared later for definitive management presented suprapubic heaviness and left flank pain for 48 hours. The diagnosis of catheter migration was confirmed by performing a computed tomography showing the tip of the catheter at the left ureterovesical junction, causing urinary flow obstruction, hydroureter, and ipsilateral hydronephrosis.

According to the cases reported so far, the diagnosis of catheter migration should be considered in a patient with long-term bladder drainage and/or urinary dysfunction who presents resistance when inflating the catheter balloon, urine leakage around the catheter or obstruction of the catheter, flank pain, urinary retention, and/or obstructive urinary symptoms.

Although most cases are reported in patients with a long-term catheter, we must bear in mind that inadvertent placement of the catheter in the ureter may occur at the first insertion.

Abdominal ultrasonography is a very useful tool to identify the catheter's location and its balloon. The computed tomography with contrast allows confirming the diagnosis of catheter migration and also allows evaluating the presence of complications such as ureteral rupture, hydronephrosis, and pyelonephritis. Patients who do not present with complications can be managed conservatively, repositioning or removing the catheter and treating urinary tract infection with antibiotics if present, as was done in this case.

## 7. Conclusion

In a patient with suprapubic cystotomy, urinary retention, hydronephrosis, flank pain, obstructive urinary symptoms, pericatheter urine leakage, or catheter blockage should make the diagnosis of catheter migration suspect. Point-of-Care Ultrasound (POCUS) examination of the urinary bladder is very helpful in detecting the hydronephrosis, the empty bladder, and the position of the catheter balloon. Patients with neurogenic bladder or long-term bladder drainage are more prone to catheter migration. The suprapubic catheter must be securely anchored to prevent the migration of the catheter tip.

## Data Availability

The data used in the review is available from the corresponding author on a reasonable request.

## Abbreviations

POCUS: Point-of-Care Ultrasound
CT: Computed tomography
ECF: Enterocutaneous fistulas
SPC: Suprapubic catheter
ED: Emergency Department.

## Additional Points

*Patient's Perspective*. I am delighted with the management I received during my suffering. I was in severe pain that I could not tolerate. I did not imagine that rare complications of my catheter would happen. I would like to appreciate the tireless efforts of the doctors and nurses who cared for me.
